# P-928. The Second Heart Program: A Peer-focused, Multidisciplinary Harm Reduction Intervention to Improve Outcomes for People Who Inject Drugs After Admission for Infective Endocarditis

**DOI:** 10.1093/ofid/ofae631.1119

**Published:** 2025-01-29

**Authors:** Alisha Atri, Robin Lennox, Timothy O’Shea, Marcie McIlveen

**Affiliations:** McMaster University, Montreal, Quebec, Canada; McMaster University, Montreal, Quebec, Canada; McMaster University, Montreal, Quebec, Canada; Mcmaster, Hamilton, Ontario, Canada

## Abstract

**Background:**

Infective endocarditis (IE) is a severe and highly prevalent injection-related infection among people who inject drugs (PWID). IE is estimated to account for 5-10% of deaths among PWID. The long-term outcomes among PWID with infective endocarditis are poor, with 5-year mortality rates over 50%. Most long-term complications are related to ongoing drug use (re-infection). However, most PWID with IE are not offered treatment for their underlying substance use disorder while in hospital. The Second Heart (SH) program is the first interdisciplinary team in Canada designed specifically to meet the unique needs of PWID with IE and which transitions with patients from the hospital into the community. The six components of the SH team are: peer support worker, systems navigator, addiction medicine physician, infectious disease physician, cardiologist/cardiac surgeon, and family physician.

**Methods:**

The Second Heart Program was a non-randomized clinical trial enrolling PWID with infective endocarditis at three tertiary care hospitals in Hamilton. It was a mixed methods study utilizing both qualitative and quantitative methods. Five quantitative surveys, two qualitative interviews, and chart review were completed over the 12-month study period.

**Results:**

Participants felt that their involvement with SH supported their physical, mental, and social recovery from IE, improved their trust in the healthcare system, and increased their engagement with their care. Out of 20 enrolled participants, all were consulted with an addictions medicine provider during initial hospitalization, 16/20 were set up with a family doctor post-discharge, 9/20 completed their 1-month follow-up survey, one participant died within 12 months of enrolment, and 12/19 completed their 12-month follow-up survey.

**Conclusion:**

The early success in this pilot project has led to the successful application of funding for an expanded version of the multidisciplinary intervention for people who have any injection-related infection, including Hepatitis and HIV.

**Disclosures:**

**All Authors**: No reported disclosures

